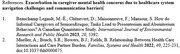# Exacerbation in Caregiver Mental Health Concerns due to Healthcare System Navigation Challenges and Communication barriers

**DOI:** 10.1002/alz70858_103898

**Published:** 2025-12-25

**Authors:** Stephanie Muskat

**Affiliations:** ^1^ Compassion in Caregiving, Toronto, ON, Canada

## Abstract

Family caregivers play a crucial role in supporting individuals with complex conditions, including neurodegenerative diagnoses, cancer, stroke, and mental health disorders. However, the demands of caregiving are often compounded by challenges in navigating healthcare systems, which exacerbate caregivers' mental health concerns. Clinicians in Ontario and Alberta who specialize in psychotherapy for family caregivers have consistently observed that difficulties with system navigation frequently surpass caregiving tasks, relationship challenges, and employment‐related stress as primary reasons for seeking mental health support.

In a private psychotherapy practice, one clinician's caseload of 22 caregiver clients over a six‐month period was analyzed to identify the primary concerns driving caregivers to seek help. Over half (54.5%) of these clients cited frustration and stress associated with navigating the healthcare system as their primary reason for seeking support. These caregivers reported increased levels of anxiety, distrust in the healthcare system, sadness, depression, shame, and burnout, reflecting the compounded toll of system inadequacies on their mental well‐being. Findings align with provincial data from Ontario which highlights rising rates of mental health challenges among caregivers and quantitative studies from Canada[1] and the US[2] which relate burden to navigating medical systems and coordinating care.

These observations underscore the inadequacies in current healthcare systems to effectively support family caregivers. Addressing these gaps requires a caregiver‐centered approach that prioritizes clear communication, validation, and a deeper understanding of caregivers’ lived experiences. Practical strategies to support caregivers must include system‐level changes, such as streamlined navigation tools, enhanced communication between providers and caregivers, and integrated supports that acknowledge and address the unique stressors caregivers face.

The findings emphasize the urgent need for system transformation to alleviate the mental health burdens associated with caregiving. By implementing caregiver‐centered care practices, healthcare systems can not only improve caregiver well‐being but also enhance the quality of care for care recipients, fostering a more sustainable and equitable healthcare environment for all stakeholders.